# Seeing Things From Different Angles – Non-Canonical Orientation Effects on Visual Word Recognition Reflect Quantitative and Not Qualitative Consequences

**DOI:** 10.1177/17470218261434511

**Published:** 2026-03-10

**Authors:** Amy Allen, Jeremy J. Tree, David Playfoot

**Affiliations:** 1 School of Psychology, Faculty of Medicine, Health and Life Sciences, University of Swansea, UK

**Keywords:** visual word recognition, letter position coding, non-canonical presentation, lexical decision

## Abstract

For skilled readers, word recognition is an apparently effortless cognitive process that can be swiftly performed across various presentation formats. A seminal study by Driver and Baylis investigated how two ‘real-world’ non-canonical orientations might disrupt visual word recognition of English words: namely, 90-degree *rotation* (e.g. the title on the spine of a book) and a vertical letter arrangement from top to bottom (e.g. the title on a building’s *marquee* banner). Driver and Baylis found that rotation had a less detrimental impact on speed and accuracy than the marquee presentation, which they interpreted as evidence for the importance of preserving a word’s ‘principal axis’. The current study seeks to replicate the findings of the original experiment and include a *canonical* (typical) presentation, which was absent in the original work. This ‘baseline’ inclusion allows for a clearer assessment of the effects of non-canonical orientations on typical lexical processing in visual word recognition. Additionally, we systematically examined the potential attenuation of these orientation manipulations on two key variables relating to word items (frequency) and nonword items (pseudohomophony) respectively. Our findings broadly replicate those of Driver and Baylis, indicating a *graded* impact of non-canonical presentation (canonical > rotated > marquee). Lexical effects remained remarkably robust across all presentation formats, suggesting that access persists even when letter strings are presented in such unfamiliar orientations. We interpret this robustness as indicating that the effects of non-canonical presentation on lexical processing are *quantitative* rather than *qualitative* in nature.

## Introduction

**Figure 1.**
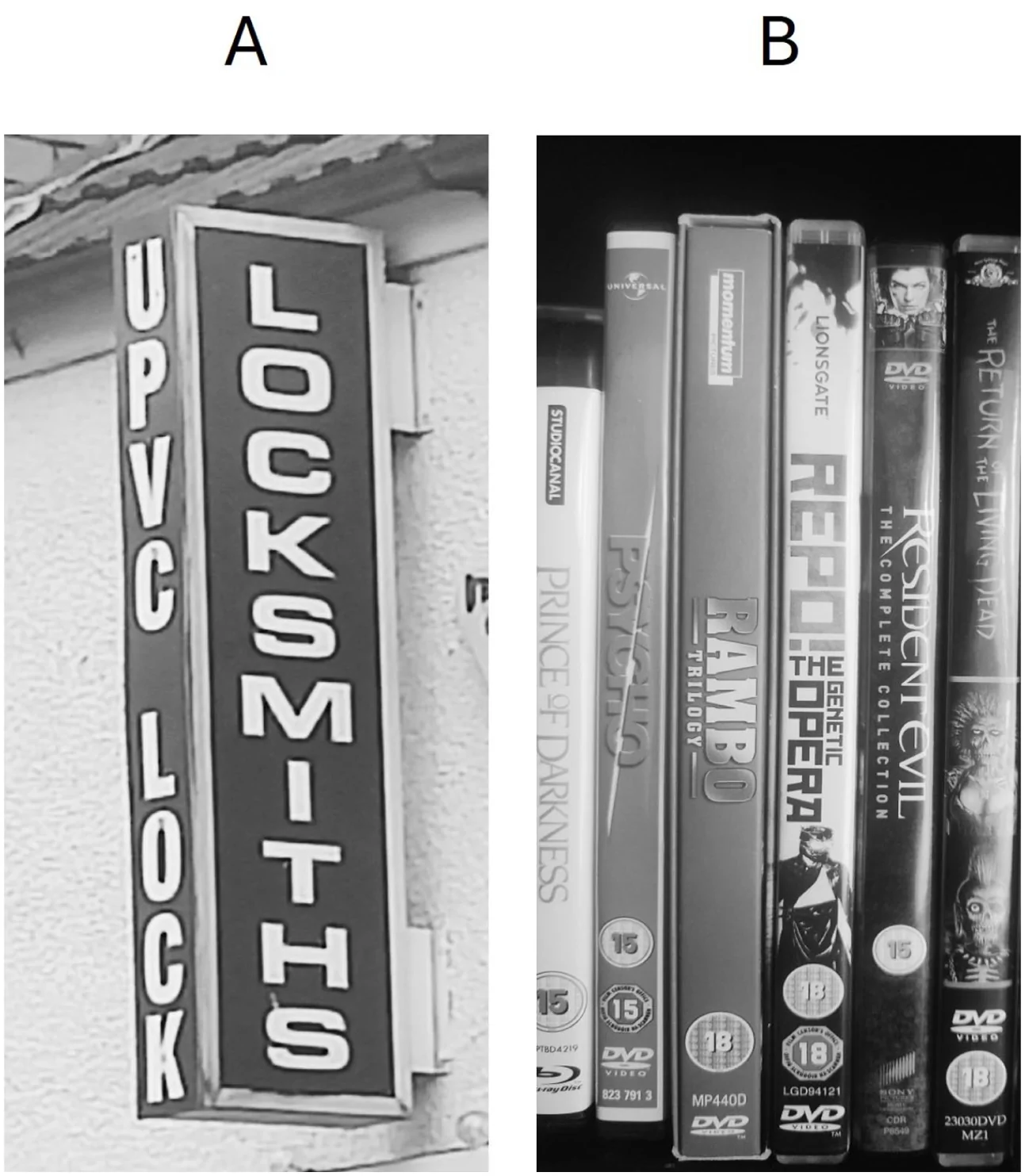
Examples of non-canonical letter orientation in the real world. Panel A shows what we refer to as ‘marquee’ presentation in the current study; Panel B shows strings rotated 90^o^ clockwise.

The skill of reading words involves the process of visual word recognition, an ability that adult readers can do both extremely quickly and accurately in a variety of contexts, which include varied presentation formats, including reading words in different fonts, CASE and sIzEs. However, most written languages have a ‘canonical’ orientation format; in English, this is the horizontal left-to-right presentation. For the most part, cognitive models of reading have been concerned with experimental manipulations that do not alter this ‘canonical’ orientation – but this belies the fact that in the ‘real world’ we often encounter familiar words in *atypical* orientations. For example, the letters on the spine of a book are often *rotated 90*^o^ (Figure 1a), and signage outside of a building can be presented from top to bottom in the *marquee* format (Figure 1b).

In order to recognise words or read them aloud, it is necessary to first identify the constituent letters and the order in which they occur. Without encoding the relative position, pairs of words that share the constituent letters (e.g. trial and trail) would be regularly confused. Cognitive models that attempt to detail how letter position is coded have largely been based on the canonical presentation, and not all non-canonical presentations of word stimuli would necessarily be predicted to affect word processing in the same way. The Local Combination Detectors, or LCD, model (Dehaene et al., 2005), for example, posits that there are neurons which, during reading acquisition, become attuned to specific and informative letter and bigram combinations. Importantly, Dehaene et al. (2005) stated that letter rotation hinders the function of letter detectors because the visual features of the letters are misidentified. Specifically, they argued that rotation beyond 40^o^ would be disruptive, but that below that threshold there would be no discernible impact. This was, in part, predicated on the work of Logothetis and Pauls (1995), who provided intracranial action potentials from primates which demonstrated tuning of visual cortex cells to viewing stimuli presented at specific (ranges of) angles within 40 to 45^o^. Presenting words in unfamiliar formats ought to disrupt the function of the local combination detectors, and hence impact the information passed on for subsequent word processing. It is also worth noting that the LCD model posits that the hierarchical organisation of the visual system would mean that groups of neurons are sensitive to increasingly broad characteristics of the stimuli, for example, moving from features to letters to bigrams. Dehaene et al. (2005) stated that bigram-level detectors would be disrupted by changes in the spacing of the letters within the bigram – in marquee presentation, the bigram is dramatically altered, and in essence, there *are* no bigrams in the word. Therefore, it could be predicted that rotating a whole word (as opposed to the individual letters within a word) should affect both the identification of individual letters and bigram detection, while marquee presentation precludes bigram detection but preserves individual letter identification.

The SERIOL model (Whitney, 2001) also predicts that letter position coding should be adversely affected by changing the orientation of the letter string that is presented. Indeed, Whitney (2002) and Gomez and Perea (2014) have argued that word processing will be slowed when stimuli are presented in rotated format because readers must first perform mental rotation so that letter position coding based on horizontal orientation can be completed. Grainger and Holcomb (2009) similarly stated that any non-horizontal alignment of letters (i.e. not only rotation but also marquee) would necessitate a transposition so that letter position coding could be completed. In other words, because current models expect that the relative position of letters within a string is coded based on visuospatial coordinates, any deviation from the orientation encountered during learning will result in poorer reading performance. These models, too, could provide a basis for predicting different impacts of rotation versus marquee presentation on the identification of letters and bigrams. We have not explored these specifically in the current study, instead focusing on how *any* non-canonical presentation disrupts written word recognition, and whether these effects are quantitative (i.e. change the speed and accuracy of processing) or qualitative (i.e. elicit a change in how processing is performed). This study aims to investigate how atypical orientation formats influence visual word recognition, as assessed through lexical decision tasks. The research focuses on understanding the effects of such non-canonical presentations on two key lexical variables established to impact word processing (the *frequency effect*) and nonword processing (the *pseudohomophone* effect, which we discuss below. In particular, we seek to revisit a seminal study on this issue (Driver & Baylis, 1995) both in an effort to replicate their key findings and to extend them further.

It is thus worth sketching out the findings of related studies that have explored the impact of non-canonical presentation of words and nonwords that are relevant to our own proposed work. As we will see, non-canonical presentation has been shown to have negative impacts on other kinds of reading tasks (such as reading speed). For example, Byrne (2002) reported that non-canonical formats (right-rotated, left-rotated and marquee formats) significantly *slowed* reading speeds (see also Yu et al., 2010) with what they dub ‘*vertical texts*’, which is the marquee format of focus in our work). In addition, the study explored another well-established phenomenon in the domain of word recognition – the *word frequency effect*. This is the observation that words encountered more frequently in a language (high frequency words) are recognised and consequently processed more quickly than less frequent words (low frequency words) – discussed further below. In this case, Byrne (2002) sought to determine whether such lexical effects are attenuated by a non-canonical format presentation (i.e. rotated 90° left/right or marquee), and surprisingly, they found no evidence for this outcome – in fact, the lexical frequency effect was *increased* for word reading speed relative to the canonical (horizontal)baseline. At the same time, reading speed showed a graded effect across the non-canonical presentations (i.e. marquee RT > rotated RT > canonical) – that is, the marquee (vertical) presentation provoked the generally slowest reading speeds.

Related work has specifically focused on the rotation presentation condition by systematically manipulating the degree of orientation change. Porter and Arblaster (2020) reported faster reading speed and higher accuracy for canonical (horizontal) orientations compared to those rotated 90° clockwise or anticlockwise (consistent with Byrne, 2002). These results also align with those of Firth et al. (2007), who systematically manipulated rotated word presentation (orientations at 15, 45 and 90°) and found that reading speed increases significantly as the text deviates further from the canonical position. Notably, there is evidence of a non-linear effect, since the slowing of reading speed becomes much more pronounced beyond a certain degree of tilt (i.e. 90° > 45 = 15°). Tree and Playfoot (2025) reported that rotated presentation affected reading accuracy in a single case of posterior cortical atrophy, with performance at 90-degree rotation being far worse than at 50-degree rotation.

The consequences of non-canonical orientation have also been investigated with respect to the *repetition priming paradigm.* In this case, the typical finding is that subsequent responses to a stimulus are faster if it (or similar) has been repeated. Benyhe and Csibri (2021) investigated the degree to which systematically rotating the prime stimulus affects the repetition priming effect on word recognition speed and accuracy. They presented a rotated prime (at various angles from 0 to 360°) followed by an upright target, measuring the time it took for participants to read the target aloud. It was found that priming was effective with rotations from 0 up to 60° from the upright position, but the effect diminished with larger rotations. Priming was strongest when the prime was presented upright, suggesting that word recognition becomes less efficient as the rotation angle increases. This particular finding is interesting in that it further supports the presence of a non-linear relationship between word recognition speed and accuracy, akin to the observations of Firth et al. (2007). Again, indicating that beyond a particular degree of orientation of rotation, the consequences of word reading speed can be much more severely disrupted.

Although the above work appears to indicate that changing presentation format can have consequences on word reading speed and accuracy, there is much less evidence on visual word recognition performance. For example, Perea et al. (2018) found that whilst the marquee presentation elicited significantly faster reaction times than the rotated presentation, the rotated presentation led to significantly fewer errors. However, this was during a masked priming lexical decision task designed to investigate how the format affects the recognition of letter identity and position – and thus perhaps less relevant to the whole word visual word recognition processes we seek to explore here. It is also important to note the absence of a canonical (horizontal) presentation – the significant implications of such an omission are discussed further below. These inconsistencies highlight the need for further investigation into whether such effects exist in a standard lexical decision paradigm that prioritises the effects of orientation alone. We note that there are several studies that have manipulated the rotation of letters within words, rotating each constituent around its own central point rather than rotating the whole word frame. Blythe et al. (2019) studied the effect of letter-in-word rotation on eye-tracking measures, again finding that rotation effects are larger when the deviation from horizontal was greater. Fernández-López et al. (2023) examined lexical decision and semantic categorisation performance with rotated letters within words. For our purposes, the key findings of those studies are that (a) rotation effects were observed and (b) the effects were larger for non-words than for words. Fernández-López et al. (2023) argued that aspects of their findings provided support for both the SERIOL model and the LCD model, indicating that further research on non-canonical word recognition is still needed.

As far as we are aware, there is only a single study (Driver & Baylis, 1995) that has systematically explored such effects of orientation change on the target items for visual word recognition in English, and thus we would argue further work is needed. Driver and Baylis (1995) undertook a key study exploring the impact of non-canonical presentation of letter strings on lexical decision performance. In their study, participants made judgements about letter strings arranged in columns with each letter upright, which we will refer to as *marquee* format, or *rotated* clockwise at a 90-degree angle (they dubbed this ‘tilted’). The focus of the work was to determine the degree to which either of these manipulations had a greater or lesser degree of interference on lexical decision performance. In particular, the focus was on a hypothesis that the ‘canonical’ orientation for English words related to a ‘principal axis’ (imagine a horizontal line from left to right running through the letter string) – this was despite the fact that no such ‘canonical’ condition was included. In any case, the simple point the authors made is that in rotating a letter string, the ‘principal axis’ is maintained, whereas this is not true for the marquee format – the prediction therefore being the latter would have a *greater disruptive* impact (akin to the findings for reading speed undertaken by Byrne, 2002). On the other hand, from the perspective of the participant viewer, the letters in the marquee remain in a ‘standard’ format (i.e. upright), which is not true for rotated items. Their key finding was consistent with the ‘principal axis’ prediction, that marquee presentation had a greater *relative* impact than tilting on speed and accuracy. Such results held true when controlling for spacing differences between formats and using alternating case to disrupt familiar global shapes, thus proving the format effect was not due to the preservation of global envelopes in the marquee format. Interestingly, the advantage of the marquee presentation became more pronounced as the letter strings increased in length, which suggests that the ‘principal axis’ plays a more vital role as the complexity of the string increases. They also considered the word frequency effect (Preston, 1935) in order to determine if this lexical variable interacted with presentation format.

We have already touched on the word frequency effect earlier in our discussion of the work on reading undertaken by Byrne (2002), but it is worth considering this variable manipulation in a bit more detail. As we have mentioned, the word frequency effect relates to the perhaps unsurprising fact that individuals process and visually recognise high frequency words much faster and more accurately than low frequency words (Gerhand & Barry, 1999; Preston, 1935, Schilling et al., 1998)

It is pertinent to consider the method by which a decision is reached during lexical decision at this point. The prevailing accounts at the time of Driver and Baylis’ (1995) work suggested that ‘word’ responses are generated when activation in the word recognition system (particularly the orthographic representations) reaches a criterion level. A ‘nonword’ response occurs when the threshold has not been reached by a deadline. Although it was published after Driver and Baylis’s paper, the Multiple Read-Out Model (Grainger & Jacobs, 1996) provided an account for lexical decisions that specified how the deadline was determined. In situations where there is a lot of overall activity in the system early in stimulus processing, the deadline is extended; the same occurs when accuracy is emphasised by task demands. It also added another mechanism by which a ‘word’ decision could be reached – a stimulus could elicit sufficient overall activation that it was *probably* a word, even if the specific representation had not been identified before the deadline. Grainger and Jacobs’ (1996) model has since fallen out of favour in the lexical decision literature based on its failure to accommodate specific participant response patterns (see Dufau et al., 2012, for a summary of these).

The more popular approach to accounting for lexical decision responses is now to apply diffusion models (Ratcliff et al., 2004; Wagenmakers et al., 2008). In a diffusion model, binary outcomes are theoretically placed on either side of a starting state on each trial. The processing of the stimulus accumulates evidence toward one of the possible outcomes and a response is elicited only when one of the outcome criteria is reached. In these models, then, there is no temporal deadline by which a response must be generated and processing continues until a decision threshold (referred to as the boundary) is reached. At its simplest, which decision is made on a single trial, and the speed with which that response is elicited, is predicated on two key parameters – drift rate and the boundary separation parameter. Drift rate is the mean rate at which evidence is accumulated toward a boundary. Note that drift is a mean rate across trials and that, because the accumulation of evidence is noisy, not every individual trial in the same condition will reach the boundary at the same latency (or necessarily reach the same boundary). The drift rate is higher when the quality of the information from the stimulus is greater. In other words, good information elicits fast responses. The ‘quality’ of information is assumed to vary from condition to condition, for example, high frequency words provide better evidence towards a ‘word’ decision than low frequency words; random letter strings provide better evidence towards a ‘nonword’ decision than pronounceable pseudowords. As detailed in Ratcliff and McKoon (2008), slower drift rates necessarily increase the likelihood of errors occurring. Boundaries can be moved relatively farther apart to emphasise accuracy (because a greater accumulation of evidence is required before a conservative decision is reached) or closer together to emphasise speed of responding. Narrower boundaries make it easier for the accumulated evidence to reach the ‘word’ decision, for example, but also require relatively less noise to accrue before the incorrect response is indicated.

Importantly for the current paper, the diffusion model also includes parameters related to processes that are not directly part of the decision itself. These non-decision components reflect encoding of the stimulus properties and the execution of the response. According to Ratcliff and Tuerlinckx (2002), across-trial variability in these non-decision components has little real effect on response distributions. However, the non-canonical presentation of the stimuli in our current experiment has a particular influence on stimulus encoding. Gomez and Perea (2014) demonstrated that rotating words beyond 45° had an effect on the non-decision parameters of a diffusion model (orientation effects were also observed for nonwords at 90° rotation). The change in presentation orientation also had an effect on drift rate. In other words, rotated stimuli slowed (a) the time to perceptually encode the stimulus and (b) the ‘quality’ of the stimulus. Finally, and crucially for the current work, large deviations from canonical presentation altered the boundary separation parameter to adopt a more conservative threshold for stimuli presented rotated at 90°. By extension, diffusion models could predict a similar pattern for marquee presentation as was observed for rotated words in Gomez and Perea’s (2014) work – difficult encoding, slower drift rate and more conservative decision boundaries, culminating in slow but accurate responding.

Driver and Baylis (1995) found the frequency effect was present regardless of presentation format (a finding that echoes that of Byrne, 2002, for word reading speed). However, much like Perea et al. (2018), unfortunately, this study did not include a ‘baseline’ condition (i.e. canonical presentation), so although it is clear that higher frequency items were responded to faster in both the non-canonical scenarios, we are not certain whether this magnitude is *proportionally smaller* than would have been typically observed with canonical items. Similarly, Driver and Baylis (1995) indicate that in general *rotated* items are faster than *marquee* items, but without a canonical baseline, we are none the wiser about the relative impact of either on the typical presentation. Put simply, the absence of a baseline condition leaves a substantial gap in the understanding of the true impact of orientation relative to typical performance levels. As a consequence, the current study sought to *replicate* this earlier work and include a baseline ‘canonical’ condition.

The inclusion of a manipulation of word frequency enables a window into the effects of non-canonical presentation for the *lexical processing* of words. However, we were also interested in manipulating a variable linked to the *non-lexical processing* of nonwords – non-lexical processing in this case referring to the rules-based conversion of orthography to phonology that allows for the naming and correct rejection of nonwords. The basic logic being, if non-canonical manipulations disrupt reading to the extent that words are not processed as words, it is likely that something similar happens to nonwords. Again, models of letter-position coding predict that informative and familiar bigrams are identified based on visuospatial coordinates (i.e. L is to the left of A). This relative position is important for the conversion from orthography to phonology when processing nonwords. In the Dual Route Cascaded model (Coltheart et al., 2001), graphemes are converted to phonemes serially. This means that inaccurate letter position identification will result in sounds being compiled in the wrong order. If non-canonical presentation disrupts letter position identification, then nonword processing will also be affected, and this should result in an observable modulation of the *pseudohomophone effect* in lexical decision. The pseudohomophone (PSH) effect, first described by Martin (1982), [Bibr bibr18-17470218261434511]refers to the finding that in lexical decision tasks, nonwords that *sound* like real words when read aloud (e.g. *SKOOL*) take longer to reject than nonwords that do not resemble real words phonologically (e.g. *ZOOL*). Although explicit theoretical explanations vary (Harm & Seidenberg, 2004; Reynolds & Besner, 2005), they generally agree that the effect reflects *lexical interference* – suggesting that it may diminish or disappear when the perception of the nonword is qualitatively altered. If we assume the PSH effect arises because phonological decoding activates a stored representation of a real word, then disrupting the conversion from orthography to phonology should *reduce* this interference. In other words, when letter strings are presented in familiar spatial canonical arrangements (e.g. standard horizontal orientation), the conflict between the spelling and sound of PSHs is likely to produce the effect. However, when the letters are presented in a non-canonical format – either rotated or presented in a marquee format – the disruption to orthographic processing may delay or interfere with grapheme-to-phoneme conversion, thereby reducing the effect.

An alternative conception of the differences in lexical decision responses between pseudohomophones and pronounceable non-words is offered by diffusion models of word recognition performance. Ratcliff et al. (2004) stated that items in lexical decision could be considered on a ‘wordness’ continuum running from highly word-like at one end to highly unlike words. Items closer to the extremes of this continuum elicit faster drift rates towards the respective decision boundaries – high frequency words are highly word-like and evidence towards the ‘word’ decision accumulates quickly; random letter strings are clearly unlike words and evidence towards the ‘nonword’ decision accumulates quickly. Under those assumptions, the difficulty that participants have in rejecting pseudohomophones stems from the fact that these are non-words that are as word-like as they can be. The principle (as described earlier) is that non-canonical presentation should result in both slower drift rates and more conservative decision boundaries (Gomez & Perea, 2014), in which case the pseudohomophone effect should remain even when stimuli are presented at different orientations – in fact it should become larger. Including both PSHs and regular nonwords in word recognition orientation studies expands the scope of investigation and provides key insights into how different visual formats influence the processing of orthographic and phonological information – forming the basis for the present study.

In summary, the primary aim of the present study is to replicate and extend the seminal work of Driver and Baylis (1995) by investigating the impact of non-canonical presentation of items in visual word recognition. Firstly, consistent with their work, we will present items in either a rotated (90°) or marquee format – whilst also critically including the canonical form of presentation as a relative baseline. Secondly, we will determine the degree to which two critical lexically driven effects: (a) word frequency for words and (b) pseudohomophone status for nonwords may be attenuated by non-canonical manipulations. Drawing on the previous work of Driver and Baylis (1995) we expect to replicate their key observations – namely that an overall advantage (shown through faster reactions times and fewer errors) will be observed for the rotated format over the marquee format during a lexical decision task and that the word frequency effect will persist in both of these formats though it remains open the degree to which this is attenuated relative to baseline.

## Method

### Participants

Overall, 92 participants signed up for the study. All participants were native English speakers enrolled on undergraduate degrees at a university in the United Kingdom. According to participant-provided demographic data, the mean age of the participants was 22.34 years (*SD* = 5.95). Sixty-two of the participants identified as female, 21 as male and 9 as non-binary. No participants reported a diagnosis of dyslexia or other language disorder. Participants were recruited via the School of Psychology’s participant pool – eligible participants were provided with a link to the online task when they signed up to the study.

### Design

The study took place within-subjects design. The key independent variable was orientation, with letter strings being presented in canonical format, rotated 90-degrees clockwise (note that this refers to the rotation of the *whole* word, not all of the individual constituent letters) or marquee. For *word* stimuli, there was a second independent variable of frequency (high vs. low); for *non-word* stimuli, the second independent variable was type (pseudohomophone vs. non-pseudohomophone). As a consequence, we ran analyses on the word trials and nonword trials separately. For both the word and the non-word analyses, the dependent variables were reaction time (in seconds) and accuracy (in proportion correct). Reaction time and accuracy provide complementary information in relation to performance in this task. Reaction time is potentially the more sensitive measure, especially given that standard deviations in accuracy are necessarily small at the high levels of performance expected in this study. Nevertheless, the accuracy of our participants may be indicative of speed-accuracy trade-offs, and reporting both reaction time and accuracy allows for clear comparison between the current study and that of Driver and Baylis (1995) in which the same dependent variables were considered.

### Materials

The lexical decision task comprised 75 high-frequency and 75 low-frequency words (taken from Weekes, 1997), along with 75 pseudohomophones and 75 non-pseudohomophonic nonwords from Howard and Best’s (1996) list. All stimuli were between four and six letters in length (thereby omitting the three letter words from Weekes’, 1997, list). Participants were presented with one of three versions of the task. Version 1 allocated a subset of 25 of each stimulus type to the canonical format, a subset of 25 to the rotated format and the remaining subset of 25 to the marquee format. Versions 2 and 3 assigned the same subsets of items to different presentation orientations, so that across all the versions of the task every stimulus had been presented at every orientation. Within each version of the task, the order in which the items appeared was randomised.

### Procedure

**Figure 2.**
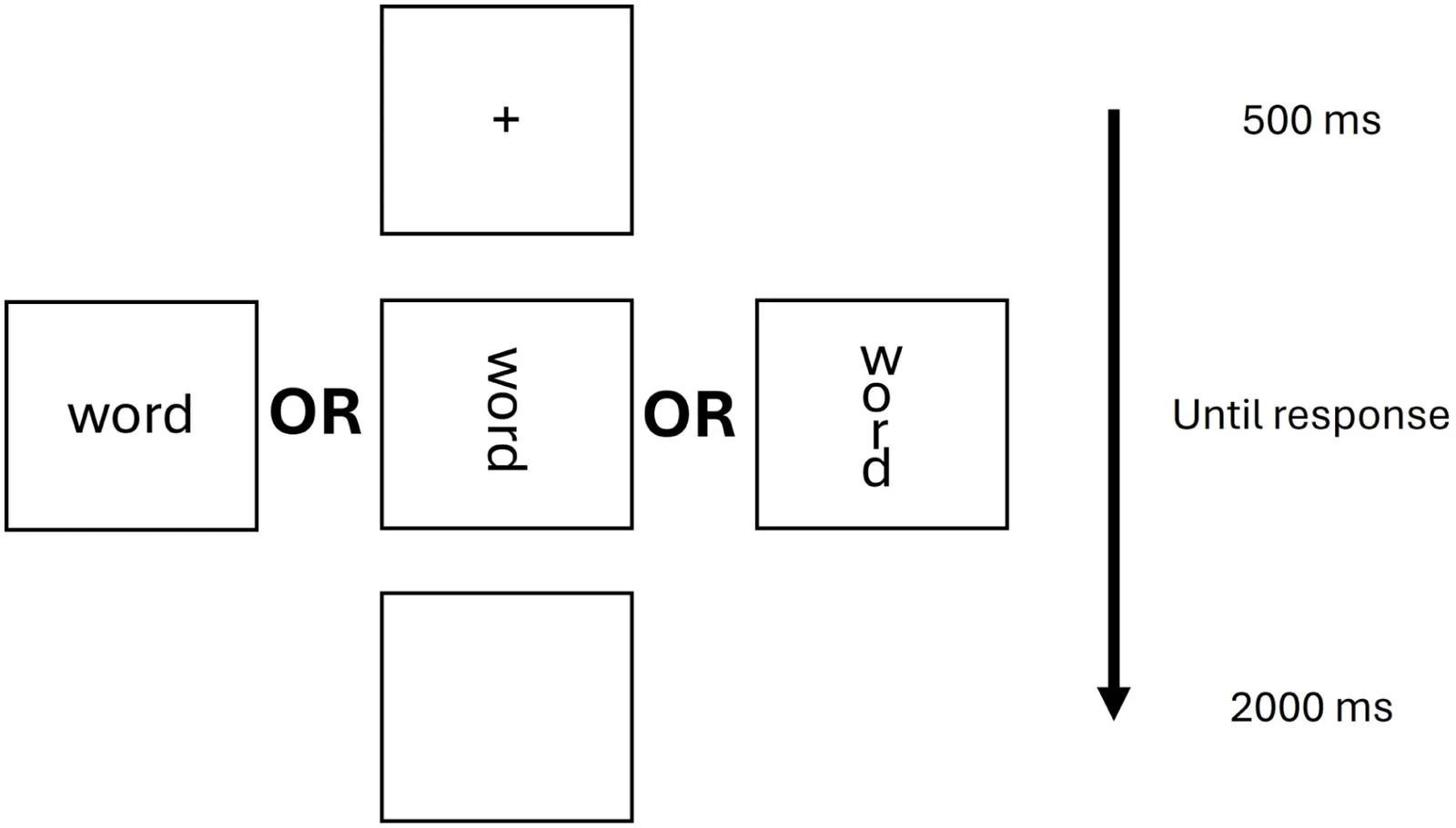
Progression of each trial, running from top to bottom. Timings are indicated in milliseconds (ms).

Participants were given instructions to complete the lexical decision task as accurately and quickly as possible, pressing the ‘L’ key if they believed the stimulus to be a real word and the ‘A’ key if they believed it was not. Within a single trial, a fixation cross was shown in the centre of the screen for 500 ms, after which the stimulus was displayed on the screen until the participant gave a response. Following this response, a blank screen appeared for 2 s before the next trial began. Figure 2 illustrates the procedure (including examples of the three different orientation conditions).

There were 300 randomised trials, with breaks after every 75 trials. During these intervals, participants were asked to press the keyboard to continue the study when they wished to do so. Stimulus presentation was controlled using PsychoPy 2 (Peirce et al., 2019) and hosted online using Pavlovia (Pavlovia.org). Stimuli were presented one at a time, using white Open Sans text on a grey background. As the study was run online, participants may have used a variety of screen sizes to complete the lexical decision task (although a standard keyboard was required, so tablets and mobile devices were disallowed). Fortunately, PsychoPy allows text size to be defined relative to the ‘height’ of the window, and hence automatically scales according to device and screen aspect ratio and are therefore the same relative size for all participants. A height of 1 is equivalent to the full height of the window; in the current experiment, letter heights were set at 0.05. Items were presented in the centre of the screen using lowercase letters. Upon study completion, a debrief form was presented containing the rationale of the study and what would happen with the information given. The study was then terminated, and participants were redirected to the original research website. An average of 20 min was needed for the entire study.

## Results

### Data Cleaning

Overall, we recruited 92 participants – a first pass examining the distribution of mean accuracies for all our participants found two individuals over three standard deviations lower than the group. As a consequence, their data were removed. A similar investigation of mean reaction times (RTs) found two participants with mean RTs which were abnormally slow (>3 SDs lower than the group mean) and thus they too were removed as outliers. Our subsequent analyses focused on the remaining 88.

### Word Analyses

#### Accuracy

As discussed earlier we sought to explore the impact of non-canonical presentation on the word *frequency effect* to replicate the seminal work of Driver and Baylis (1995). In Table 1, we present the observed and estimated marginal means for our various conditions along with 95% confidence intervals. In this case, we were interested in seeing the degree to which the standard frequency effect may be *attenuated* by our different non-canonical presentations, with the inference that if this ubiquitous lexical effect were eliminated this likely constitutes a *qualitative* change to the perceived stimulus. However, Table 1 appears to suggest the frequency effect occurs for accuracy performance in all cases, and our subsequent analyses will explore this further.

**Table 1.** Observed Means and EMMs for Accuracy Across Frequency and Presentation Conditions.

Presentation	Frequency	Raw mean	Raw 95% CI	EMM	EMM 95% CI
Canonical	High	0.96	[0.95, 0.97]	0.98	[0.97, 0.99]
	Low	0.87	[0.85, 0.88]	0.94	[0.91, 0.96]
Rotated	High	0.93	[0.92, 0.94]	0.96	[0.95, 0.98]
	Low	0.84	[0.83, 0.86]	0.92	[0.89, 0.94]
Marquee	High	0.91	[0.89, 0.92]	0.95	[0.93, 0.96]
	Low	0.84	[0.83, 0.86]	0.92	[0.89, 0.94]

*Note.* Raw means represent the empirical accuracy rates and the EMMs are estimated marginal means from the GLMM model. Confidence intervals (CIs) are based on 95% coverage. EMMs = Estimated Marginal Means; GLMM = generalised linear mixed model.

A generalised linear mixed model (GLMM) was conducted to examine the effects of Frequency and Presentation on response accuracy with participants included as a random effect. The model was specified with Frequency and Presentation as fixed effects, forming a 2 (Frequency: High, Low) × 3 (Presentation: Canonical, Marquee, Rotated) factorial design. Both *Participant* and *Item* were included as random effects to account for individual and item-level variability in responses. The GLMM for accuracy employed a binomial distribution with a logit link function. Our analyses revealed a main effect of Frequency, χ^2^(1) = 14.66, *p* < .001, indicating overall accuracy of responses was generally higher for High Frequency items (HF = 0.968, LF = 0.925). There was also a significant main effect of Presentation, χ^2^(2) = 64.77, *p* < .001 (Canonical = 0.966, Marque = 0.935, Rotated = 0.945), and a significant interaction between Frequency and Presentation, χ^2^(2) = 24.61, *p* < .001, which we further explore below. If we focus on the main effect of Frequency across presentation conditions (see Table 1) we see that this was significant in all three presentations instances: (a) Canonical High/Low Frequency (estimate = −0.046, 95% CI [−0.024, −0.068], *SE* = 0.011, *z* = −4.12, *p* < .001 [Holm-adjusted]), (b) Marquee High/Low Frequency (estimate = –0.028 [0.001, –0.058], *SE* = 0.015, *z* = –1.908, *p* = .05 [Holm-adjusted]) and (c) Rotated High/Low Frequency (estimate = –0.046 [–0.017, –0.074], *SE* = 0.014, *z* = –3.173, *p* = .003 [Holm-adjusted]). As a consequence, it seems the lexical frequency effect on observed accuracy scores is pervasive regardless of presentation condition.

**Figure 3.**
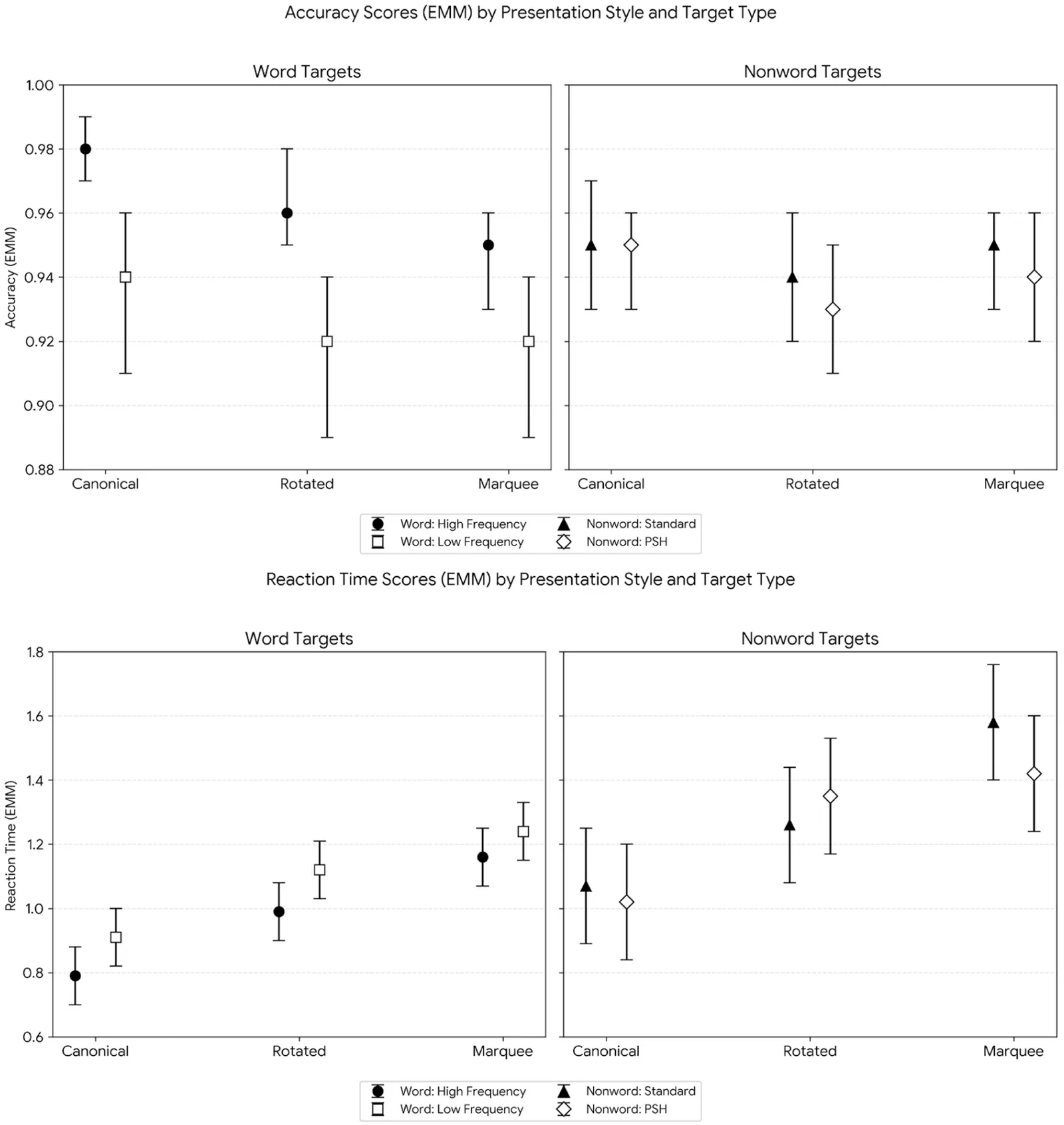
EMM for accuracy (top) and reaction time (bottom) by condition. Error bars represent 95% confidence intervals. *Note.* EMMs = Estimated Marginal Means.

Moving to the observation of the different presentation formats across word Frequency type, we undertook planned comparisons across presentation conditions. Firstly, for the High Frequency items we found an accuracy pattern consistent with a *graded effect* of Canonical *>* *Rotated* *>* *Marquee*: (a) relative to our ‘baseline’, accuracy was significantly higher for Canonical versus Rotated (estimate = 0.019, 95% CI [0.01, 0.029], *SE* = 0.005, *z* = −3.916, *p* < .001 [Holm-adjusted]), and for Canonical versus Marquee (estimate = 0.034 [0.02, 0.048], *SE* = 0.007, *z* = 4.868, *p* < .001 [Holm-adjusted]) and (b) across the two atypical orientations, accuracy was superior for Rotated versus Marquee (estimate = −0.015 [0.004, 0.026], *SE* = 0.006, *z* = 2.721, *p* = .007 [Holm-adjusted]). Secondly, for the Low Frequency items, we found a slightly different pattern: (a) relative to our ‘baseline’ accuracy was significantly higher for Canonical versus Rotated (estimate = 0.019 [0.004, 0.033], *SE* = 0.007, *z* = 2.534, *p* = .034 [Holm-adjusted]), and for Canonical versus Marquee (estimate = 0.017 [0.003, 0.031], *SE* = 0.007, *z* = −2.31, *p* = 0.042 [Holm-adjusted]) and (b) across the two atypical orientations, accuracy was effectively identical, Rotated versus Marquee (estimate = −0.002 [−0.017, 0.012], *SE* = 0.007, *z* = −0.296, *p* = .767 [Holm-adjusted]). Data relating to all analyses, including those described below, are presented in Figure 3.

Overall, our analysis of words in this case yielded two key findings: (1) the word frequency effect on accuracy was ubiquitous across all presentation types and (2) the effect of presentation type varies across the frequency types; for High Frequency items there was a *graded* effect of presentation type (Canonical > Rotated > Marquee), whilst for Low Frequency was clearly generally attenuated, and the pattern of poorer performance of the two atypical orientations relative to baseline only. The inclusion of a baseline provided an important indicator in that it demonstrated that atypical orientation does generally decrease accuracy, even though across Rotated/Marquee the pattern changes depending on the frequency of the targets.

#### Reaction Times

A linear mixed-effects model, again including both participants and items as random effects, was conducted to examine the effects of Frequency and Presentation on reaction times. The model was specified with Frequency and Presentation as fixed effects, forming a 2 (Frequency: High, Low) × 3 (Presentation: Canonical, Marquee, Rotated) factorial design. The analysis revealed a significant main effect of Frequency, *F*(1, 146) = 14.80, *p* < .001, indicating generally faster mean RTs for High Frequency versus Low Frequency items across the board (see Table 2). As before, there was also a significant main effect of Presentation, *F*(2, 13041) = 85.75, *p* < .001, (estimated marginal means: Canonical = 0.849, Rotated = 1.201 and Marquee = 1.055) and planned contrasts demonstrated the same graded effect observed for accuracy *Canonical < Rotated < Marquee* (see Table 2) – (a) relative to our ‘baseline’, reaction time was significant faster for Canonical versus Rotated (estimate = 0.206, 95% CI [0.153, 0.259], *SE* = 0.027, *z* = 7.617, *p* < .001 [Holm-adjusted]) and for Canonical versus Marquee (estimate = 0.352 [0.299, 0.405], *SE* = 0.027, *z* = 13.034, *p* < .001 [Holm-adjusted]) and (b) across the two atypical orientations reaction time was also significantly different, with Rotated faster than Marquee (estimate = 0.146 [0.093, 0.199], *SE* = 0.027, *z* = 5.416, *p* < .001 [Holm-adjusted]). Finally, unlike for accuracy, there was no significant interaction between Frequency and Presentation *F*(2, 13141) = 0.348, *p* = .706.

**Table 2.** Observed Means and EMMs for Reaction Time (in Seconds) Across Frequency and Presentation Conditions.

Presentation	Frequency	Raw mean	Raw 95% CI	EMM	EMM 95% CI
Canonical	High	0.79	[0.75, 0.83]	0.79	[0.70, 0.88]
	Low	0.91	[0.88, 0.94]	0.91	[0.82, 1.00]
Rotated	High	0.99	[0.96, 1.03]	0.99	[0.90, 1.08]
	Low	1.12	[1.03, 1.21]	1.12	[1.03, 1.21]
Marquee	High	1.16	[1.10, 1.22]	1.16	[1.07, 1.25]
	Low	1.24	[1.17, 1.32]	1.24	[1.15, 1.33]

*Note.* Raw means represent the empirical reaction times and the EMMs are estimated marginal means from the GLMM model. Confidence intervals (CIs) are based on 95% coverage. EMMs = Estimated Marginal Means; GLMM = generalised linear mixed model.

In sum, both RT and accuracy analyses demonstrated that the Frequency effect was observed across all presentation formats, with little evidence of any kind of attenuation in either case. The inclusion of the baseline condition demonstrated that both Rotated and Marquee presentations result in slower and less accurate performance, but that Marquee is generally associated with even worse performance than Rotated (consistent with the work of Driver & Baylis, 1995 and Byrne, 2002 for word reading). However, this pattern was not observed for accuracy with low-frequency items.

### Nonword Analyses

#### Accuracy

A GLMM was conducted to examine the effects of Nonword type and Presentation on response accuracy. The model was specified with Nonword type and Presentation as fixed effects, again both *Participant* and *Item* as random effects and employing a binomial distribution with a logit link function, forming a 2 (Nonword type: NW, PSH) × 3 (Presentation: Canonical, Marquee, Rotated) factorial design. This determined a non-significant main effect of Nonword type, χ^2^(1) = 0.324, *p* = .569, and a significant main effect of Presentation type, χ^2^(2) = 10.242, *p* = .006 (Canonical = 0.95, Rotated = 0.937 and Marquee = 0.945). Planned contrasts demonstrated a different pattern to that often observed with the word targets – (a) relative to our ‘baseline’, accuracy was significant higher for Canonical versus Rotated estimate = –0.013, 95% CI [−0.022, −0.005], *SE* = 0.004, *z* = −2.998, *p* < .008 (Holm-adjusted), but not for Canonical versus Marquee estimate = −0.005 [−0.013, 0.003], *SE* = 0.004, *z* = −1.269, *p* = .204 (Holm-adjusted) and (b) across the two atypical orientations accuracy was also not significantly different; Rotated versus Marquee estimate = −0.008 [ −0.016, 0.002], *SE* = 0.027, *z* = −1.906, *p* = .113 (Holm-adjusted). Finally, there was no interaction between Nonword type and Presentation (χ^2^[1] = 0.697, *p* = .706).

**Table 3.** Observed Means and EMMs for Proportion Accuracy Across Nonword Type and Presentation Conditions.

Presentation	Type	Raw mean	Raw 95% CI	EMM	EMM 95% CI
Canonical	Nonword	0.91	[0.90, 0.92]	0.95	[0.93, 0.97]
	PSH	0.90	[0.88, 0.91]	0.95	[0.93, 0.96]
Rotated	Nonword	0.90	[0.88, 0.91]	0.94	[0.92, 0.96]
	PSH	0.87	[0.86, 0.89]	0.93	[0.91, 0.95]
Marquee	Nonword	0.90	[0.89, 0.91]	0.95	[0.93, 0.96]
	PSH	0.89	[0.88, 0.90]	0.94	[0.92, 0.96]

*Note.* Raw means represent the empirical accuracy rates and the EMMs are estimated marginal means from the GLMM model. Confidence intervals (CIs) are based on 95% coverage. PSH = pseudohomophone; EMMs = Estimated Marginal Means; GLMM = generalised linear mixed model.

In summary, for correct rejection accuracy of Nonwords we did not see an often-reported effect of pseudohomophony in this case. Interestingly, the typical *graded effect* of orientation seen for word accuracy was *not* present for nonword accuracy – with some evidence that *rotated* presentation was the worst performer. However, we are cautious to make much of this observation given the generally very small observed differences (see Table 3).

#### Reaction Time Analyses

A linear mixed-effects model was conducted to examine the effects of Nonword type and Presentation on reaction times (Table 4) with participants and items included as random effects. The model was specified with Nonword type and Presentation as fixed effects, forming a 2 (NW type: Pseudohomophones, NWs) × 3 (Presentation: Canonical, Marquee, Rotated) factorial design. The analysis revealed that there was no significant main effect of nonword type, *F*(1, 13176) = 0.425, *p* = .51, but there remained a significant main effect of Presentation, *F*(2, 13176) = 20.462, *p* < .001 (Canonical = 1.045, Rotated = 1.303 and Marquee = 1.503), and again the interaction between Condition and Presentation was not significant, *F*(2, 13176) = 1.48, *p = *.228. As before we further explored the main effect of presentation and determined that: (a) there was a significant difference between the Canonical and Rotated conditions, estimate = 0.258, 95% CI [0.117, 0.398], *SE* = 0.072, *z* = 3.593, *p* < .001 (Holm-adjusted), (b) a significant difference between the Canonical and Marquee conditions, estimate = 0.458 [0.317, 0.598], *SE* = 0.072, *z* = 6.38, *p* < .001 (Holm-adjusted) and (c) a significant difference between Rotated and Marquee conditions, estimate = 0.20 [0.059, 0.341], *SE* = 0.072, *z* = 2.787, *p* = .005 (Holm-adjusted).

**Table 4.** Observed Means and EMMs for Reaction Time (in Seconds) Across Nonword Type and Presentation Conditions.

Presentation	Type	Raw mean	Raw 95% CI	EMM	EMM 95% CI
Canonical	Nonword	1.07	[0.97, 1.16]	1.07	[0.89, 1.25]
	PSH	1.02	[0.97, 1.08]	1.02	[0.84, 1.20]
Rotated	Nonword	1.26	[1.22, 1.30]	1.26	[1.08, 1.44]
	PSH	1.35	[1.17, 1.53]	1.35	[1.17, 1.53]
Marquee	Nonword	1.58	[1.33, 1.84]	1.58	[1.40, 1.76]
	PSH	1.42	[1.33, 1.51]	1.42	[1.24, 1.60]

*Note.* Raw means represent the empirical reaction time data and the EMMs are estimated marginal means from the GLMM model. Confidence intervals (CIs) are based on 95% coverage. EMMs = Estimated Marginal Means; GLMM = generalised linear mixed model.

Overall, it seems that the key graded effect of presentation format is observed in speeds of responses for *both* words and nonwords. The effect of pseudohomophony was not present in either case for this experiment and there was no evidence that this non-lexical variable interacted with presentation format.

## General Discussion

As mentioned earlier the current study was an attempt to replicate the work of Driver and Baylis (1995) and improve things by including a baseline and two different experimental manipulations targeting lexical and non-lexical processing: namely word frequency and nonword pseudohomophony, where only the former was previously investigated. In that seminal work, they reported two key things. Firstly, for reaction times, items presented in a rotated (tilted) format were always faster than marquee (upright), regardless of the lexical status of the strings (i.e. words or nonwords) or the frequency of the words. For accuracy, the pattern was a little different in that a similar rotated > marquee pattern was observed but only for words. Unfortunately, their mean scores were not reported, and although median scores were provided this was only via a figure in the manuscript – making direct comparison to our observed values impossible. However, a casual inspection of the figure in their publication also highlights quite high error rates – ~10% for high frequency and ~20% for low frequency items and it is not clear why that should be the case (perhaps a few extremely poor performers in their *N* = 30 sample). We are naturally reluctant to assume it may reflect differing population literacy levels since our study and theirs use undergraduate psychology students.

In any case, our work appears to generally replicate the findings of Driver and Baylis (1995) with respect to the impact of the presentation variable, but our inclusion of a canonical condition clarifies that for visual word recognition, non-canonical presentation is generally worse than a ‘typical’ baseline (replicating the work of Byrne, 2002 in their observation of reading speeds). Critically the inclusion of this baseline enables us to interpret the pattern as a *graded* effect (canonical > tilted > marquee), the consequences of which are true for the speed of processing of both words and nonwords (directly replicating the work of Driver & Baylis, 1995 — albeit with a better powered study in our case). The story for accuracy in our case was a little different, in that this same graded pattern was (a) most obviously observed for accuracy for *high frequency items* and (b) less apparent with nonwords, with a hint that rotation may have proportionally greater impact relative to baseline (though we would suggest caution in that interpretation). Nonetheless, as commented earlier, it is apparent that errors rates were generally much higher in the original study which may explain our divergent findings. There was no clear indication of a speed-accuracy trade-off. We note that there are nuances to non-canonical presentation with regard to real-world experience. Books commonly appear on shelves such that their titles appear rotated, as do DVDs, video games and other physical media. In this regard, seeing text rotated 90^o^ clockwise is more common than seeing marquee presentation. There are therefore differences in the familiarity with the presentation format itself which may influence the ease and efficiency of the mental reconfiguration of the stimulus for word recognition. That said, when encountering marquee presentation in the real world, there are some words that are much more likely to appear in that format (e.g. restaurant, hotel, open). It may be that processing of non-canonical word presentation is affected by lexical familiarity, format familiarity, and by an interaction of the two. This remains an open empirical question.

The fact that we observed an interaction between frequency and presentation in accuracy, but not in reaction time, is interesting. Ratcliff and McKoon (2008) argued that these two variables should be strongly linked, with slower drift rates necessarily resulting in longer RTs and a higher likelihood of errors in diffusion models. This interaction in one dependent variable and not the other is at odds with that position. We do not wish to overstate this discrepancy given that it was an unexpected finding, and we did not have an a priori reason to predict that one dependent variable would be more informative than the other. Nevertheless, it is worth outlining some potential mechanisms that may be at play. Grainger and Jacobs (1996) proposed that there were three decisions that could be reached in lexical decision tasks. The first was to locate an exact match for the stimulus in the lexicon (eliciting a ‘word’ response), which is achieved more quickly when the stimulus is high frequency. The second is predicated on the overall level of activation in the lexicon exceeding a threshold where a stimulus is *probably* a word even if an exact match hasn’t been identified. The third is that a time-limit is reached before an exact match is found, and before global lexical activation is sufficient to suggest that the stimulus might be a word (i.e. a ‘nonword’ response). They argued that the time limit was impacted by task demands, rather than on an item-by-item basis. In word trials, therefore, an incorrect response is given when the deadline is reached before an exact match is located, or enough global lexical activation is accumulated, and hence a real word is presumed to be a nonword. Whitney (2002) and Grainger and Holcomb (2009) have argued that non-canonically presented stimuli must be reorganised before word processing can begin. Our data show that participants take longer to respond correctly to items presented in the marquee format than in the rotated format, which presumably reflects a difference in the ease or efficiency of the reorganisation of the stimulus onscreen. Under the assumptions of Grainger and Jacobs (1996), this impacts how much time is left before the decision deadline is reached (i.e. there is less of a ‘window’ in which to search the lexicon in marquee trials than in rotated trials) and increases the likelihood of a word being incorrectly rejected. Combined with the fact that, all else being equal, a low frequency word is also slower to accumulate lexical activation than a high frequency word, it is possible to explain why there is a significant difference between accuracy in marquee and rotated conditions for high frequency, but not low frequency, words. Recognising a high frequency word that was presented in marquee format is harder than in rotated format because there is a tighter deadline following reorganisation, but the rearranged stimulus is familiar enough that it can be accessed in time. In contrast, because low frequency words take longer to reach a recognition threshold, there is insufficient time remaining following both rotation and marquee presentation – a floor effect of sorts. Subsequent deadline models have allowed for the deadline to be adjusted on a trial-by-trial basis as well. The Leaky Competing Accumulator model (Dufau et al., 2012), for example, adjusts response thresholds according to the accuracy on the previous trial so that repeated success in making lexical decision responses speeds up subsequent responses until an error is made. Under those circumstances, reaction time is affected by the frequency at which errors are made. This could potentially offer an explanation for the interaction in accuracy but not reaction time – the number of mistakes made would impact the accuracy dependent variable but the number of trials between mistakes would alter the RT. However, we are unable to examine this possibility in the current dataset because participants were not provided with accuracy feedback during the task, and hence we cannot analyse fluctuations in RT across the experiment.

Although deadline models have fallen out of favour, they do highlight the potential role that decision criteria may have in explaining our results. Gomez and Perea (2014) demonstrated that non-canonical presentation can influence both drift rate and the boundary separation parameter of a diffusion model, such that extreme deviation from the canonical format can result in the adoption of conservative decision thresholds. This would result in slower, more accurate responses. It is not clear how a change in boundary separation could result in the patterns observed in our data, especially given that reaction time and speed are closely related in diffusion models. That said, it is worth noting that there was no interaction between presentation format and word frequency in the behavioural data reported by Gomez and Perea (2014), so their analyses of the effects of the independent variables on the parameters of their model would be unlikely to account for such an interaction in any case. Nevertheless, that stimulus presentation has been shown to affect both the decision and non-decision components of lexical decision making does provide a potential avenue for future research.

In addition, our findings overlap with those of Driver and Baylis (1995) in that we also observed a ubiquitous frequency effect both for speed and accuracy measures, which with the inclusion of a baseline condition in our case, were reasonably commensurate with typical (canonical) word processing (unknown in their study). However, we did not observe any effects of pseudohomophony for nonwords. We would argue that the fact that lexical effects did not appear to interact with these non-canonical presentation formats must indicate that access to some form of representation of the whole word still occurs. It is also important to note that we observed differences in RT that followed the pattern of canonical < rotated < marquee. This pattern is interesting as it pertains to letter position coding. Witzel et al. (2011) showed that transposed-letter priming effects can be observed in marquee presentation, which they argued could be accommodated by assuming that letter position coding was ordinal (first letter, second letter) rather than visuospatial (leftmost letter, one-from-the-leftmost letter). Their findings could equally be accounted for by assuming a mental transposition of the non-horizontal stimulus to allow for the coding of visuospatial coordinates (Grainger & Holcomb, 2009). We argue that our findings (and those of Witzel et al., 2011) are more readily accommodated by the latter account. In the rotated condition, the word as a whole matches the relative position of letters once it has been mentally rotated back to horizontal; in the marquee condition the stimulus needs to be more extensively reconfigured. We suggest that the rotation takes less time than the reconfiguration, but that after that initial process has been completed the word recognition proceeds as it does for canonically presented words. We mentioned, in the introduction, that the LCD and SERIOL models (Dehaene et al., 2005, and Whitney, 2001, respectively) would predict different impacts of rotation versus marquee presentation on the identification of individual letters and of bigrams. While our study was not designed to specifically manipulate letter- or bigram-level variables, it *does* provide evidence that there is an additional reaction time cost for marquee presentation over and above word rotation. Precisely how this occurs is an open question but does not appear to be easily accommodated under existing models.

In their original study, Driver and Baylis (1995) considered whether the effects of word frequency might interact with presentation format, suggesting that high-frequency words – being highly familiar – may also be more strongly associated with *canonical presentation* (see Besner, 1989; Haber & Haber, 1981). This led to the prediction that disrupting this familiarity through non-canonical formats might have some attenuation effect. However, their study lacked a canonical baseline condition, making it difficult to assess this claim directly. Our study addressed this limitation by including such a baseline and attempting a broader replication and a larger sample size. Importantly, we did observe a pattern somewhat consistent with their prediction, but only for accuracy, and only in a graded way – the inclusion of our baseline condition clearly demonstrated that speed of processing appears to be universally impacted by non-canonical presentation. With this graded pattern of disruption confirmed and the additional observation that lexical access must persist, we are left with the question: why might rotated presentation still prove superior to marquee presentation? In their original work Driver and Baylis (1995) argued for a ‘*role of principle axis of letter strings in providing a reference frame to which component letters are described*’, with the inference that so long as this ‘principle axis’ is maintained there will be an information processing advantage. We might well accept this suggestion, with one important caveat, which is that severe disruption to such a ‘principle axis’, argued to be the case with the marquee format, does not qualitatively change word perception sufficient to massively attenuate classic lexical effects such as frequency for words – *lexical access must persist regardless.*
